# Preclinical Validation of Talaporfin Sodium-Mediated Photodynamic Therapy for Esophageal Squamous Cell Carcinoma

**DOI:** 10.1371/journal.pone.0103126

**Published:** 2014-08-04

**Authors:** Shinya Ohashi, Osamu Kikuchi, Mihoko Tsurumaki, Yukie Nakai, Hiroi Kasai, Takahiro Horimatsu, Shin'ichi Miyamoto, Akira Shimizu, Tsutomu Chiba, Manabu Muto

**Affiliations:** 1 Department of Therapeutic Oncology, Kyoto University Graduate School of Medicine, Kawahara-cho, Shogoin, Sakyo-ku, Kyoto, Japan; 2 Department of Gastroenterology and Hepatology, Kyoto University Graduate School of Medicine, Kawahara-cho, Shogoin, Sakyo-ku, Kyoto, Japan; 3 Institute for Advancement of Clinical and Translational Science, Kyoto University Hospital, Kawahara-cho, Shogoin, Sakyo-ku, Kyoto, Japan; MGH, MMS, United States of America

## Abstract

Photodynamic therapy (PDT) kills cancer cells via a photochemical reaction mediated by an oncotropic photosensitizer. Herein, we performed an experimental preclinical study to validate the anti-tumour effect of talaporfin sodium-mediated PDT (t-PDT) for esophageal squamous cell carcinoma (ESCC) cells. We used human ESCC cells derived from various differentiation grades or resistant to 5-fluorouracil (5-FU). The cytotoxic effect of t-PDT was determined by evaluating cell viability, apoptosis and generation of reactive oxygen species (ROS) and DNA double-strand breaks. Furthermore, the anti-tumour effect of t-PDT was assessed using an anchorage-independent cell-growth assay and xenograft transplantation models. t-PDT induced potent cytotoxicity in ESCC cells independent of their differentiation grade or 5-FU resistance. Moreover, t-PDT induced robust apoptosis, as indicated by cell shrinkage, perinuclear vacuolization, nuclear fragmentation and induction of annexin V-positive cells. This apoptotic response was accompanied by concurrent activation of ROS, and induction of DNA double-strand breakage. Importantly, t-PDT suppressed efficiently anchorage-independent cell growth as well as ESCC-xenografted tumor formation. In aggregate, t-PDT showed anti-tumor potential for ESCC cells with various histological grades or chemoresistance, providing a novel translational rationale of t-PDT for the treatment of ESCC.

## Introduction

Photodynamic therapy (PDT) is a light-based oncological intervention that uses a tumor-specific photosensitizer and laser irradiation [1]. Briefly, the administration of a tumor-targeting photosensitizing agent followed by irradiation with a specific wavelength generates reactive oxygen species (ROS) that cause DNA damage, resulting in a selective anti-tumor effect [2]. The first clinical trial of PDT was reported by Dougherty *et al* [3], the US Food and Drug Administration approved it as the first drug–device combination [4]. Thereafter, PDT has been used in the treatment of a wide range of cancers, including breast [3], skin [5], lung [6], head and neck [7], [8] and gastric [9] cancer.

Regarding esophageal diseases, the efficiency of PDT using some photosensitizers such as a mTHPC (metatetrahydroxyphenylchlorin) or a porfimer sodium has been demonstrated in the treatment of superficial esophageal cancer [10], [11] or the prevention of the development of adenocarcinoma from high-grade dysplasia in Barrett's esophagus [12]. In addition, we reported that PDT with porfimer sodium is quite useful as a salvage treatment for ESCC patients with local failure after definitive chemoradiotherapy [13]. However, PDT with the first-generation photosensitizer including porfimer sodium has some major problems, such as a high risk of skin phototoxicity and a need for a large and expensive excimer dye laser system [13].

Recently, a new photosensitizer, talaporfin sodium, and a diode laser system have been developed as a second-generation PDT to circumvent the above-mentioned problems [14]. Talaporfin sodium features rapid clearance from the skin. In addition, the absorption wavelength of talaporfin sodium (664 nm) is longer than that of porfimer sodium (640 nm) [14]. Therefore, theoretically, it is expected to have a low rate of phototoxicity and to be effective on deep tissue layers. Indeed, talaporfin-mediated PDT (t-PDT) exhibited lower skin phototoxicity in a clinical trial for early lung cancer [15]. Furthermore, the diode laser systems are much more convenient devices in terms of size and cost of equipment compared with the excimer dye laser systems.

Thus, t-PDT with a diode laser may function as an ideal combination among the PDT drugs and devices that are available for the treatment of ESCC. However, an experimental preclinical study remains to be performed to validate the efficacy of t-PDT for ESCC. Here, we determined the cytotoxic effects of t-PDT in culture and xenograft transplantation models using ESCC cells representing various differentiation grades and resistance to 5-fluorouracil (5-FU), which is a key chemotherapeutic drug for ESCC [16]. Moreover, we explored the mechanisms underlying the anti-tumor effect of t-PDT.

## Materials and Methods

### Cell lines and cell culture

The human ESCC cell lines TE-5 (derived from poorly differentiated ESCC), TE-8 (derived from moderately differentiated ESCC), TE-10 (derived from highly differentiated ESCC) and TE-11 (derived from moderately differentiated ESCC) were obtained from the Riken BioResource Center (Ibaragi, Japan) [17]. TE-5R and TE-11R cells, which are derived from TE-5 and TE-11 cells, respectively, are 5-FU-resistant ESCC cells that were established originally by us via exposure of parental cells to gradually increasing concentrations of 5-FU. TE-5R and TE-11R cells were 15.6-fold or 7.9-fold resistant to 5-FU compared with parental cells, respectively (manuscript in preparation). Of note, TE-11R cells are highly transformed cells with advanced anchorage-independent cell-growth activities, as well as tumorigenicity. Accordingly, we used mainly TE-11R cells to verify the cytotoxic or anti-tumor effect of t-PDT. All ESCC cells were cultured in RPMI1640 medium (Life Technologies Corp., Grand Island, NY, USA), supplemented with 10% fetal bovine serum (FBS) (Life Technologies Corp.), 100 µg/mL streptomycin and 100 U/mL penicillin (Life Technologies Corp.).

### Photosensitizer and laser light delivery system

Talaporfin sodium was obtained from Meiji Seika Pharma Co., Ltd (Tokyo, Japan) [18]. The laser we used in this study was a diode laser system using a semiconductor laser irradiator (Panasonic Healthcare Co., Ltd., Yokohama, Japan) [18]. The details of the settings in the laser system as well as the optimal doses of talaporfin sodium and laser irradiation *in vitro* and *in vivo* were referred to the previously reports [18], [19].

### Measurement of fluorescence intensity in ESCC cells treated with talaporfin sodium

To show the uptake of talaporfin sodium in cultured ESCC cells, we measured the fluorescence intensity of talaporfin sodium. Cells were treated with the indicated concentrations of talaporfin sodium for 24 h. Cells were washed twice with phosphate-buffered saline (PBS), immersed in 2% FBS/PBS without talaporfin sodium, and then they were followed by the measurement of the mean fluorescence intensity per 10000 cells by flow cytometer (BD LSRFortessa Flow Cytometer; BD Biosciences, San Jose, CA, USA), which excites at 640 nm with emissions in the range of 670±14 nm.

### Talaporfin-mediated PDT *in vitro*

ESCC cells were placed into 96-well plates at a concentration of 1×10^4^ cells per well, and incubated with talaporfin sodium (0–100 µg/mL) for 24 h, Cells were washed twice with PBS, immersed in fresh medium without talaporfin sodium, and then they were subjected to laser irradiation (wavelength, 664 nm; laser power, 15 mW/cm^2^; total amount of irradiation, 10 J/cm^2^) [18]. Phase-contrast images were acquired using a Nikon Eclipse TE300 microscope (Nikon Instruments Inc., Tokyo, Japan). Cell viability at 48 h after t-PDT was assessed using the Cell Proliferation Reagent WST1 assay (Roche Applied Science, Penzberg, Germany).

### Annexin V/Propidium iodide double-staining flow cytometry

The FITC Annexin V Apoptosis Detection Kit I (BD Biosciences) was used to assess cell apoptosis induced by t-PDT. Cells (TE-11R) were harvested at 4 h after treatment with talaporfin sodium with or without subsequent irradiation, and were stained with annexin V–FITC and propidium iodide (PI). These cells were analysed with flow cytometer (BD FACSCanto II Flow Cytometer; BD Biosciences). Unstained cells were used as negative controls. Data collected were analysed using the BD FACSDiva software (BD Biosciences). Cells were discriminated into four groups: viable cells (annexin V–/PI–), necrotic dead cells (annexin V–/PI+), early apoptotic cells (annexin V+/PI–) and late apoptotic cells (annexin V+/PI+) [20].

### Measurement of intracellular ROS levels

The generation of intracellular ROS during t-PDT was measured using an OxiSelect Intracellular ROS assay kit (Cell Biolabs, Inc., San Diego, CA, USA), which uses the oxidation-sensitive fluorescent probe 2′,7′-dichlorodihydrofluorescein diacetate (DCFH-DA). This assay was performed by adding DCFH-DA to TE-11R cells 4 h after t-PDT and quantifying intracellular ROS levels by detecting oxidized fluorescent 2′,7′-dichlorodihydrofluorescein (DCF) using a fluorometric plate reader (ARVO X5; PerkinElmer, Waltham, MA, USA) at 480/530 nm.

### DNA double-strand breakage assay

Phosphorylation of the histone H2A variant (γ-H2AX) is a marker of DNA double-strand breaks, which is the gravest form of DNA damage [21]. We investigated the DNA double-strand breaks induced during t-PDT using the OxiSelect DNA Double-Strand Break Staining Kit (Cell Biolabs, Inc.). Cells (TE-11R) were treated with the indicated talaporfin sodium with or without subsequent irradiation, and were stained with an anti-phospho-histone antibody 24 h after treatment. DNA double-strand breaks labeled with FITC-conjugated secondary antibody were assessed using a fluorescence microscope (BZ-9000 BIOREVO, Keyence Corp., Osaka, Japan).

### Soft agar colony-formation assays

The inhibitory effect of anchorage-independent cell growth after t-PDT was examined by soft agar colony-formation assays. Briefly, 2.5×10^4^ cells of TE-11R cells were suspended in 0.67% agarose containing media with or without talaporfin sodium (30 µg/mL), and overlaid on top of 1% agarose containing the medium per well. Subsequently, the gel was laser irradiated 24 h after the gel formation, and cells were grown for 2 weeks. Colonies were stained with 0.02% Giemsa Stain Solution (Muto Pure Chemicals Co., Ltd., Tokyo, Japan), and the number and the size of colonies per high-power field were measured using a Nikon Eclipse TE300 microscope.

### Xenograft transplantation and t-PDT *in vivo*

All experiments conformed to the relevant regulatory standards and were approved by the Institutional Animal Care and Use Committee of the Bozo Research Center (Approval number: APS13003, APS13006) (Tokyo, Japan). Mice were bred and housed in a temperature- and light-controlled facility with unlimited access to food and water. Xenograft transplantation using ESCC cells was performed as described previously [22]. Briefly, 10×10^6^ TE-11R cells were suspended in 50% matrigel (BD Biosciences), followed by their subcutaneous implantation into the dorsal skin of NOD/SCID male mice (7 weeks of age; CLEA Japan, Inc., Tokyo, Japan). Xenografted tumors were used for t-PDT when they had reached a tumor volume of about 50–150 mm^3^ at 35 days after the injection. Tumors were free of evident necrosis at the time of treatment. The optimal doses of talaporfin sodium and laser irradiation were as reported previously [19]. In brief, the indicated concentration of talaporfin sodium (0–10 mg/kg) was administered intravenously via the tail vein of NOD/SCID mice, and tumors were irradiated using a semiconductor laser irradiator at a light dose of 100 J/cm^2^ and a wavelength of 664 nm 2 h after the injection of talaporfin sodium. The tumor volume was monitored for 21 days. Mice were painlessly sacrificed under the appropriate anesthesia with carbon dioxide inhalation and the cervical dislocation.

### Histological analyses and immunostainings

The tumors were resected, fixed with 4% buffered paraformaldehyde solution, embedded in paraffin, and sectioned into 4-µm thickness. For the histological evaluation, the sections were stained with hematoxylin and eosin (H&E). For the immunohistochemistry, the sections were immunostained as previously described [23]. In brief, the sections were incubated with the primary antibody, a mouse monoclonal antibody Ki67 antigen (NCL-Ki67-MM1, Novocastra Laboratories, UK), at 4°C overnight, after which the secondary antibodies were added. Negative controls were prepared with isotype IgG.

### Statistical analyses

Statistical analyses were performed using the SPSS statistics software (version 17; SPSS Inc., Chicago, IL, USA). Data from triplicate experiments are presented as the mean ± standard deviation (S.D.) and were analysed by a 2-tailed paired *t*-test. Two-way repeated-measures ANOVA with a post-hoc Bonferroni correction was used for multiple comparisons with a control group. *P*<0.05 was considered statistically significant.

## Results

### Measurement of talaporfin sodium in cultured ESCC cells

**Figure 1:**
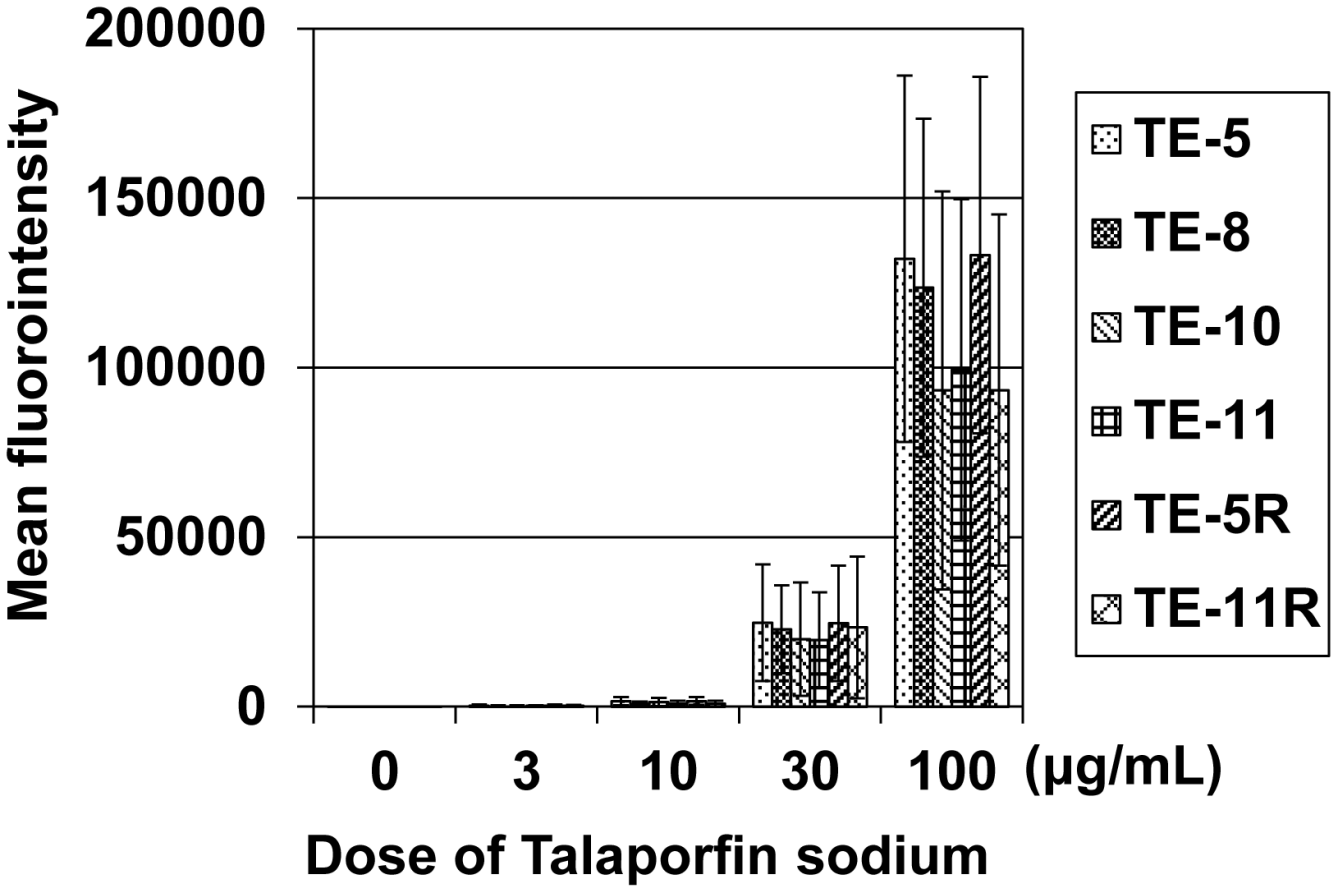
The fluorescence intensity of talaporfin sodium in cultured ESCC cells. The fluorescence intensity of talaporfin sodium in cultured ESCC cells (10000 cells) incubated with the indicated concentrations of talaporfin sodium for 24 h was measured by flow cytometry. As shown, talaporfin sodium was almost equally incorporated to the various ESCC cells *in vitro*.

Uptake of talaporfin sodium by cultured ESCC cells was determined by the fluorescence intensity of talaporfin sodium. As shown in Fig. 1, talaporfin sodium was incorporated in ESCC cells in a dose-dependent manner (Data S1 in File S1). Cell type did not affect the incorporation of talaporfin sodium (Fig.1, Data S1 in File S1).

### Cytotoxic effect of t-PDT in ESCC cells

**Figure 2:**
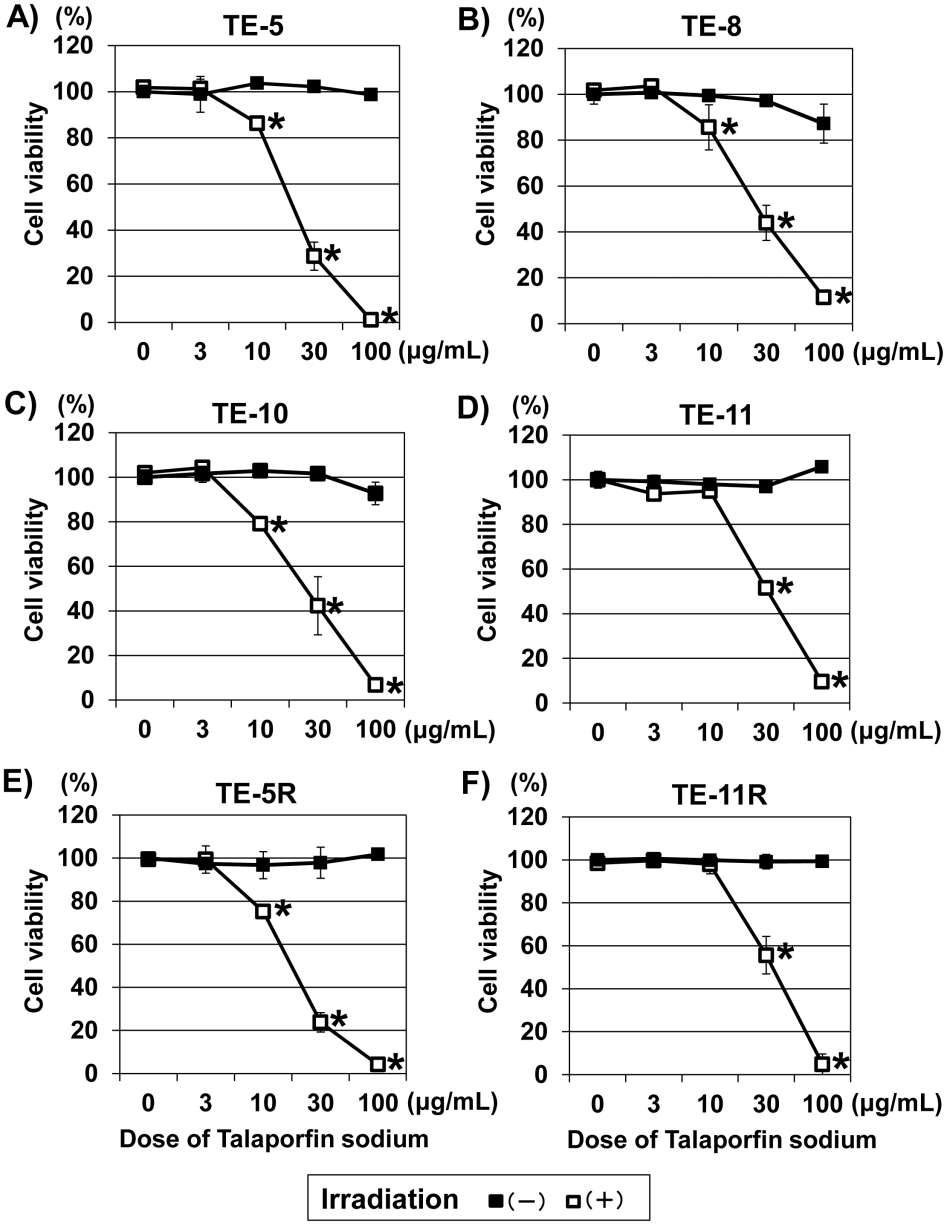
Cytotoxic effect of t-PDT on ESCC cells. Cell viability at 48-PDT was assessed using the WST-1 assay. t-PDT induced a talaporfin sodium dose-dependent cell death in ESCC cells (white square in the figures) regardless of differentiation grade or 5-FU resistance. (A) TE-5 (derived from poorly differentiated ESCC), (B) TE-8 (derived from moderately differentiated ESCC), (C) TE-10 (derived from highly differentiated ESCC), (D) TE-11 (derived from moderately differentiated ESCC), (E) TE-5R (5-FU-resistant cells derived from parental TE-5 cells) and (F) TE-11R (5-FU-resistant cells derived from TE-11 cells). A viability of 100% was defined as the amount of absorption at 450 nm found in untreated (non-irradiated and absence of treatment with talaporfin sodium) cells. Each point represents the mean ± S.D. from experiments conducted at least in triplicate. *P<0.01 vs untreated (non-irradiated and absence of treatment with talaporfin sodium) cells (*n* = 3).

To determine the cytotoxic effect of t-PDT in ESCC cells, we examined cell viability using a WST-1 assay 48 h after t-PDT. As shown in Fig. 2, neither talaporfin sodium alone nor diode laser alone exhibited cytotoxicity in ESCC cells; however, the combination of talaporfin sodium with subsequent laser irradiation induced an apparent dose-dependent cytotoxicity. Moreover, those cytotoxic effects were almost equally observed in ESCC cells independent of the grade of differentiation (Fig. 2 A–D) and 5-FU sensitivity (Fig. 2E and F) (Data S2 in File S1).

### Induction of apoptosis by t-PDT in TE-11R cells

**Figure 3:**
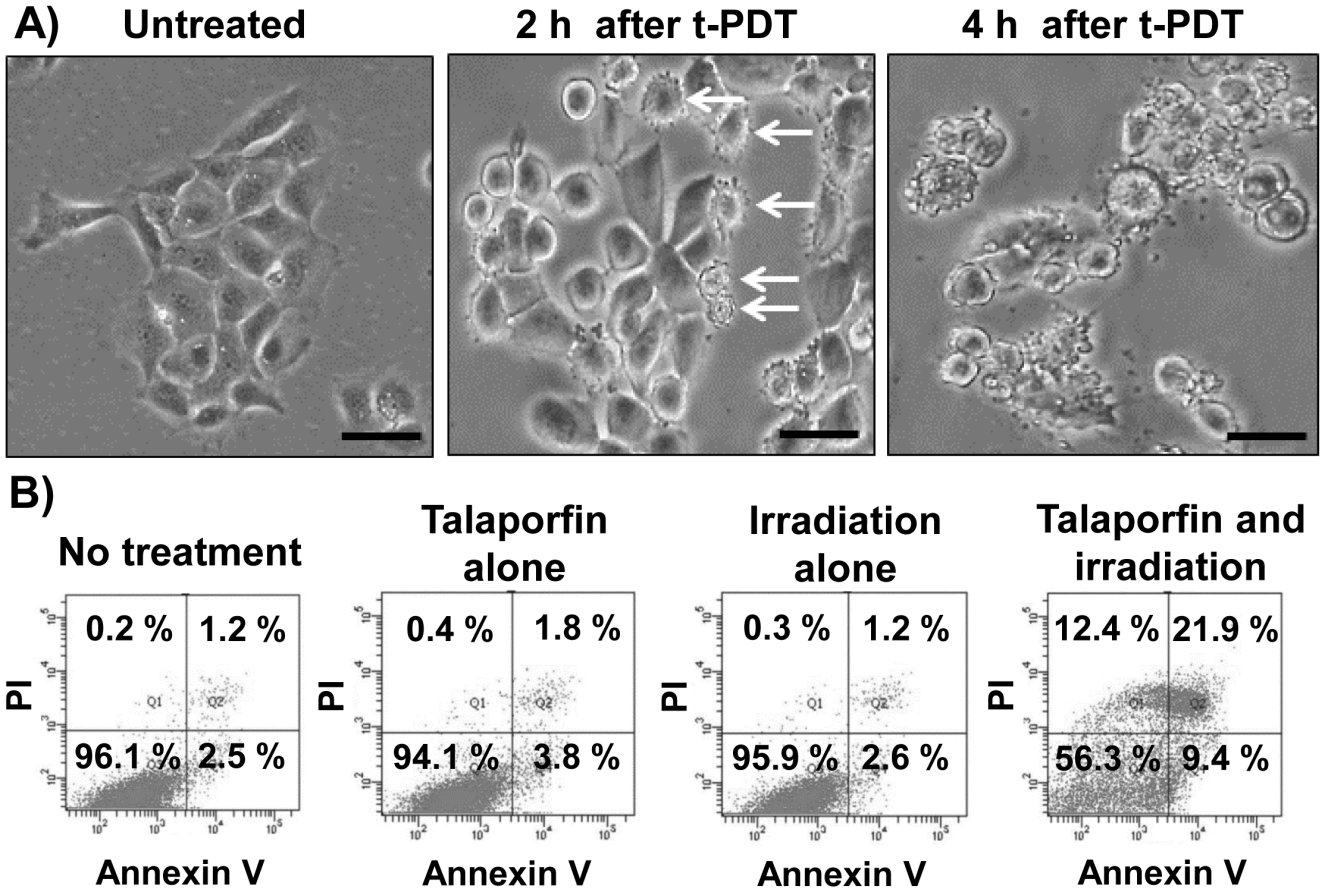
Induction of apoptosis in TE-11R cells treated with t-PDT. (A) TE-11R cells were pretreated with talaporfin sodium (30 µg/mL) for 24 h, and then irradiated (10 J/cm^2^). Phase-contrast images were taken at 2 or 4 h after t-PDT. The images of untreated cells are also shown. Arrows indicate perinuclear vacuolization and cell shrinkage suggesting apoptosis. Scale bar, 100 µm. (B) Flow cytometric analysis of apoptosis in TE-11R cells treated with or without talaporfin sodium (30 µg/mL) for 24 h with or without subsequent laser irradiation (10 J/cm^2^). Cells were stained with FITC-labelled annexin V and propidium iodide (PI) 4 h after irradiation. A representative experiment out of three is shown.

We assessed the morphological changes over time in ESCC cells treated with t-PDT. Fig. 3A shows the phase-contrast images of TE-11R cells treated with t-PDT, which demonstrates that perinuclear vacuolization and cell shrinkage were robustly induced within 2 h of laser irradiation. Moreover, nuclear fragmentation and disruption of the cell membrane were observed 4 h after treatment. Thus, drastic morphological changes that were indicative of apoptosis were observed. Moreover, the number of annexin V-positive cells was increased 4 h after t-PDT, whereas treatment with talaporfin alone or irradiation alone had no effect (Fig. 3B, Data S3 in File S1).

### Increased levels of intracellular ROS and induction of DNA double-strand breakage by t-PDT

**Figure 4:**
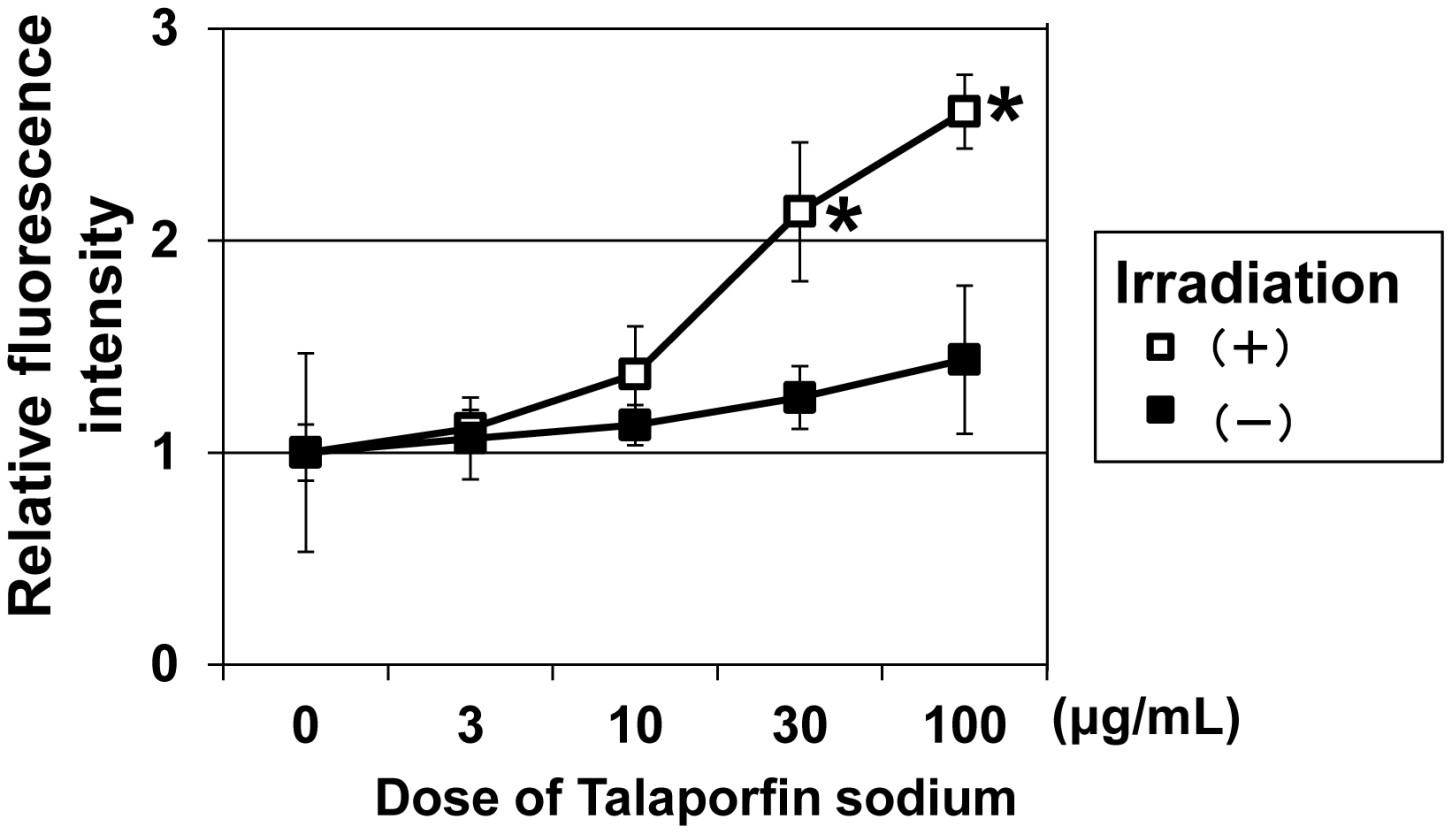
Generation of ROS in TE-11R cells treated with t-PDT. (A) TE-11R cells were treated with the indicated concentrations of talaporfin sodium for 24 h and received irradiation subsequently. Intracellular ROS levels at 4 h after irradiation treatment were determined by DCF assay. The intracellular ROS level was significantly increased by t-PDT in a talaporfin sodium dose-dependent manner. * *P*<0.01 vs untreated (non-irradiated and absence of treatment with talaporfin sodium) cells (n = 3).

**Figure 5:**
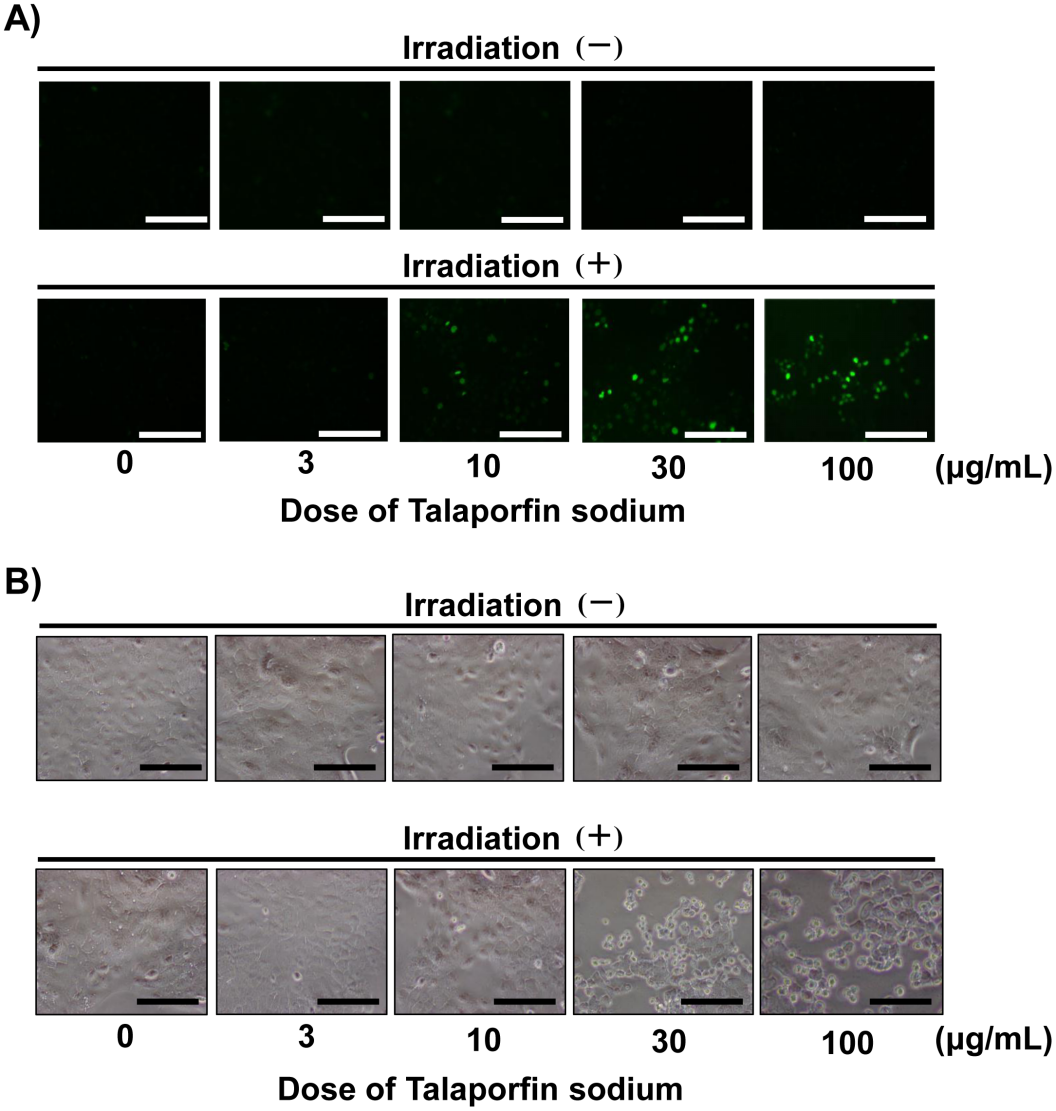
Formation of DNA double-strand breaks in TE-11R cells treated with t-PDT. TE-11R cells were treated with the indicated concentrations of talaporfin sodium for 24 h with or without subsequent laser irradiation (10 J/cm^2^). (A) The expression of γ-H2AX was evaluated by fluorescence microscopy at 24 h after the irradiation. Under the non-irradiated conditions, γ-H2AX expression was not observed (upper panels), whereas a talaporfin sodium dose-dependent phosphorylation of γ-H2AX was found in the irradiated groups (lower panels). A representative experiment out of three is shown. Scale bar, 50 µm. (B) Phase contrast image was shown at 24 h after the irradiation. Scale bar, 50 µm.

Next, we examined whether ESCC cells treated with t-PDT show increased levels of intracellular ROS or DNA damage. As shown in Fig. 4, a DCF assay revealed that intracellular ROS levels were significantly elevated by t-PDT in a talaporfin sodium dose-dependent manner. Furthermore, talaporfin sodium induced a dose-dependent phosphorylation of γ-H2AX in TE-11R cells treated with t-PDT, indicating that t-PDT induced DNA double-strand breaks, which is the most serious type of DNA damage (Fig. 5).

### t-PDT blocked anchorage-independent cell growth of TE11R cells

**Figure 6:**
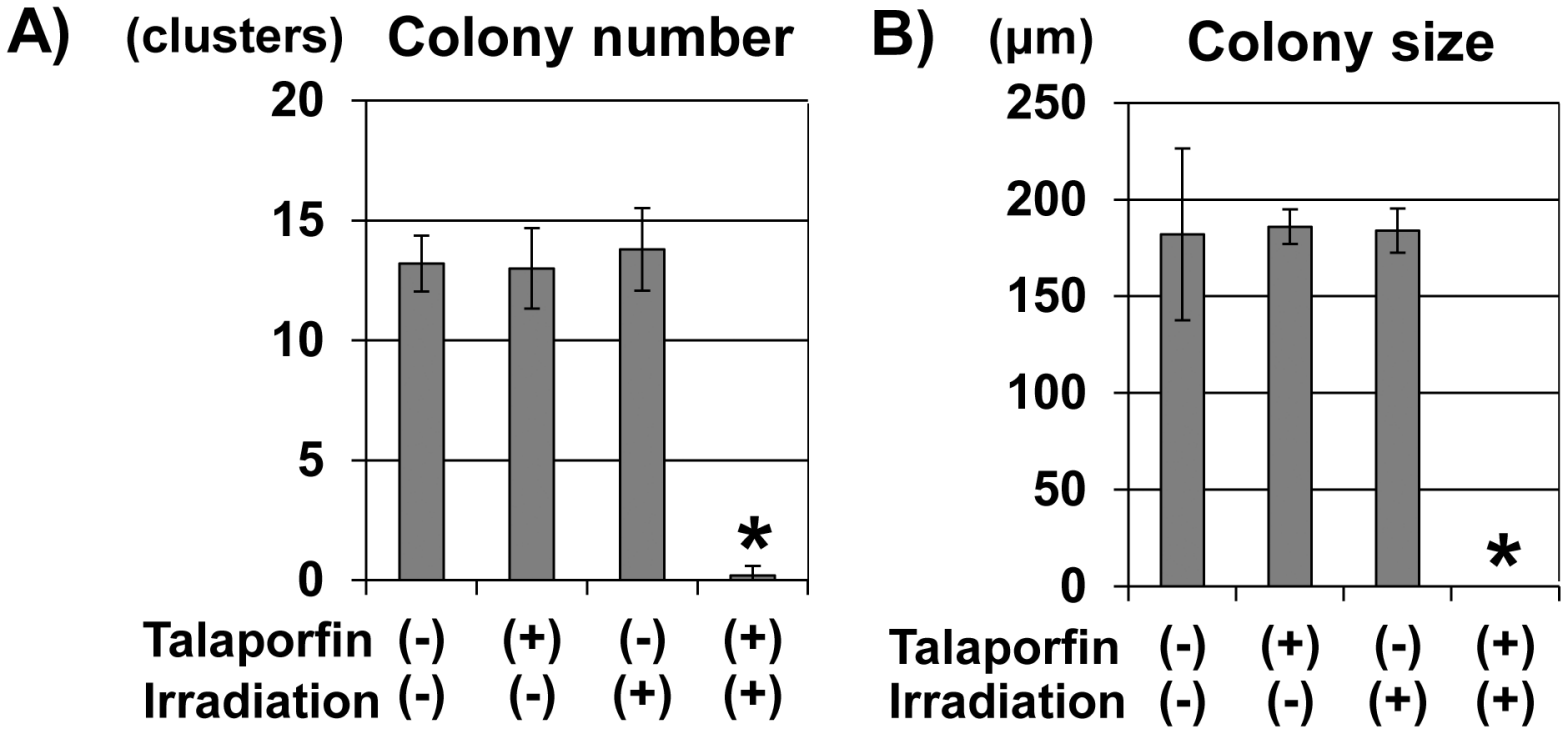
Inhibition of anchorage-independent cell growth due to t-PDT. Soft-agar colony-formation assays demonstrated the activity of anchorage-independent cell growth in TE-11R cells. The histograms show the average number (A) or size (B) of colonies per high-power field. Assays were performed in triplicate. t-PDT blocked colony formation completely, although talaporfin sodium alone or irradiation alone had no effect. *P<0.01 vs untreated (non-irradiated and absence of treatment with talaporfin sodium) cells (n = 3).

We tested whether t-PDT affects the anchorage-independent cell growth of ESCC cells. Consistent with the data showing the cytotoxic effect of t-PDT (Fig. 1), neither talaporfin sodium alone nor diode laser alone influenced colony formation; however, t-PDT completely blocked colony formation (Fig. 6).

### t-PDT suppressed the ESCC-xenografted tumors

**Figure 7:**
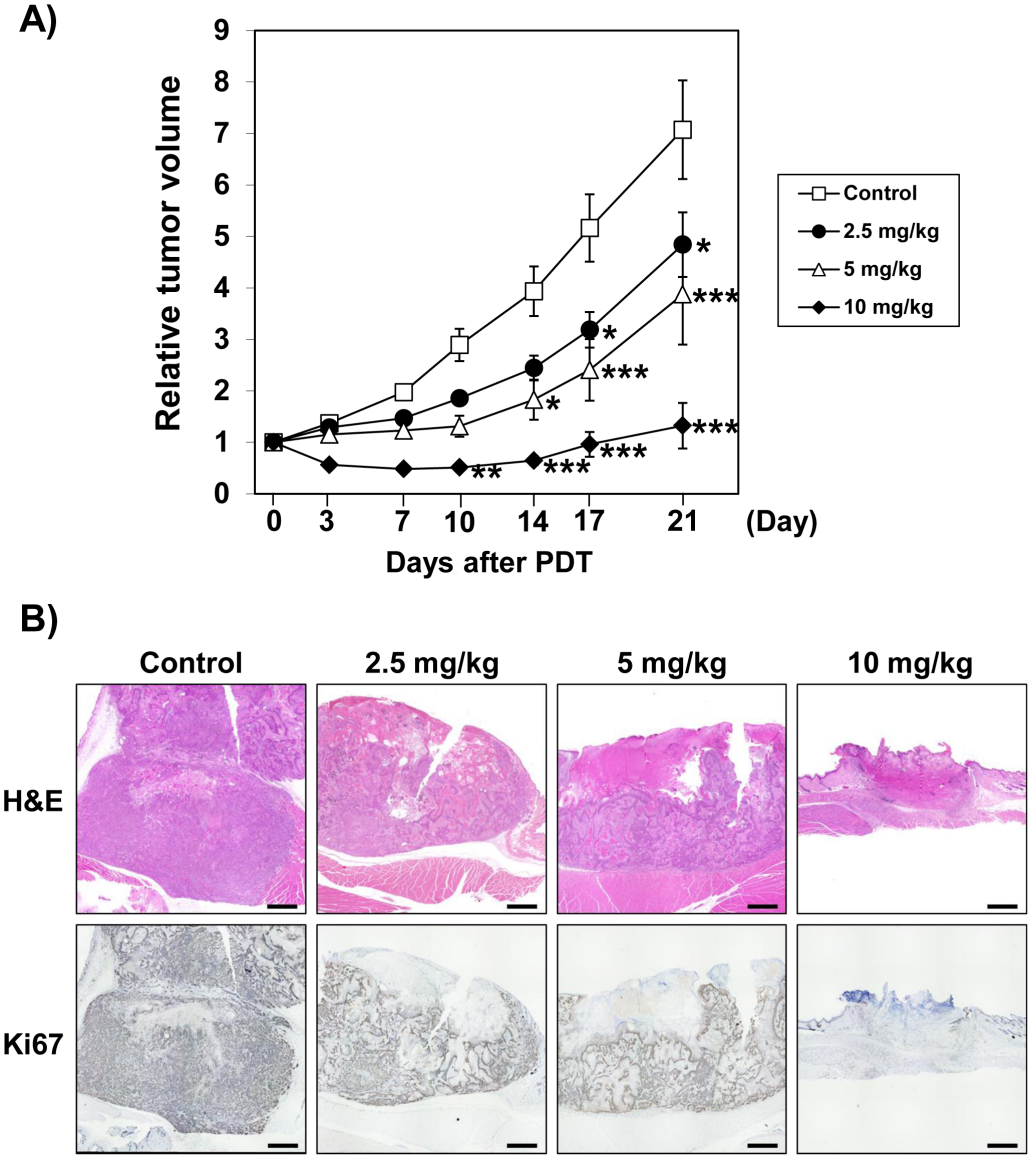
t-PDT suppress tumor formation *in vivo*. (A) Tumor response in mice treated with t-PDT at the indicated doses of talaporfin sodium. t-PDT was performed when tumor volume reached about 50–150 mm^3^. Each point represents the average response rate (relative tumor volume on tumor size in day 0) of seven mice. Talaporfin sodium dose-dependent tumor reduction was shown in xenografted ESCC tumors treated with t-PDT. *P<0.01, **P<0.001, ***P<0.0001 vs the control groups at the indicated time points (n = 7). (B) Hematoxylin and eosin and immunohistochemical (Ki67) staining. Scale bar, 1mm. Ki67 positive cells indicate the viable and proliferative ESCC cells.

Lastly, we examined the anti-tumor effect of t-PDT in ESCC-xenografted tumors in NOD/SCID mice. t-PDT successfully suppressed tumor growth *in vivo* in a talaporfin sodium dose-dependent manner (Fig. 7A, Data S4 in File S1). There was no significant change in body weight between the groups. Damage to normal skin was not observed in any of the mice. Significant tumor regrowth was not evident over the 3 weeks that followed t-PDT at the dosage of 10 mg/kg of talaporfin sodium. Histopathological and immunohistochemical examination revealed that the tumors irradiated after the administration of talaporfin sodium were subjected to the potent tissue injury, which was accompanied with completely abolished Ki67 staining (Fig. 7B).

## Discussion

In this study, we demonstrated that t-PDT induced potent cytotoxicity in ESCC cells independent of their differentiation grade or 5-FU sensitivity. Apoptotic cells were induced within 4 h after t-PDT and were accompanied by increased levels of intracellular ROS and DNA double-strand breaks. Moreover, t-PDT suppressed anchorage-independent cell growth in ESCC cells *in vitro*, and, most importantly, showed a potent anti-tumor effect in ESCC cells *in vivo*.

ESCCs are heterogeneous tumors with highly differentiated cell nests known as central keratinization (i.e., keratin pearl) and/or poorly differentiated cell nests [22]. Histological grade regarding ESCC differentiation is associated with functional malignant potentials, such as invasion and metastasis [24], [25],_ENREF_28 and with poor prognosis [24]. In this study, t-PDT yielded potent cytotoxicity in ESCC cells derived from both highly differentiated and poorly differentiated histological grade. Moreover, it exhibited a valid cytotoxic effect in 5-FU-resistant ESCC cells, among which TE-11R cells showed an undifferentiated and proliferative phenotype as well as resistance to 5-FU (unpublished data). Thus, a cytotoxic effect of t-PDT in ESCC cells can be expected, regardless of histological grade or the presence or absence of resistance to 5-FU.

The anti-tumor effect of t-PDT has been reported as being mediated by multiple cell death pathways, such as apoptosis or necrosis, depending on the treatment intensity and/or tumor properties [26]. In this study, apoptotic morphological changes were found within 4 h after t-PDT. Annexin V-positive cells were consistently induced by t-PDT. However, PI-positive cells indicating necrosis were also detected in our experiment; thus, both apoptotic and necrotic pathways appear to be involved in the cytotoxicity observed in ESCC cells. Although the specific effectors that discriminate these various cell death pathways were not identified in this study, we presented clear evidence of the potent cytotoxicity and anti-tumor effect of t-PDT in ESCC cells based on *in vitro* and *in vivo* studies, respectively.

We demonstrated that t-PDT induced an increase of intracellular ROS levels, as well as DNA double-strand breaks in ESCC cells. Our data are consistent with previous reports that PDT-induced cytotoxicities are mediated by the generation of ROS [27]_ENREF_33 or DNA double-strand breaks [28]. Thus, the induction of those factors is suggested to be related to the promotion of cell death, which leads to the active tumoricidal response of t-PDT in ESCC cells.

Anchorage-independent cell growth or tumor formation in xenograft transplantation is the hall-mark of transformed cells, which is the most well-established *in vitro* or *in vivo* assay to detect the malignant transformation of the cells [29]–[31]. In the present study, we demonstrated that t-PDT efficiently inhibited anchorage-independent cell growth, as well as tumorigenicity in ESCC cells. These results suggest that t-PDT successfully eliminates transformed cells with highly malignant potentials.

Taken together, our results demonstrate that t-PDT had a direct anti-tumor effect in ESCC cells. Although previous studies have revealed the inhibitory effects of some ROS inhibitors [32] or caspase-specific inhibitors [33] on the action of t-PDT, the anti-tumor effects of t-PDT cannot be explained only by a direct cytotoxic action through ROS generation or apoptosis, because secondary vascular effects through endothelial damage are also closely associated with the anti-tumor mechanisms of t-PDT *in vivo* [26], [34]. Those indirect anti-tumor effects of t-PDT in ESCC cells warrant elucidation in further studies.

In conclusion, we demonstrated the promising effect of t-PDT in ESCC via experimental preclinical studies. We are currently evaluating the clinical efficacy of t-PDT as a salvage treatment for patients with local failure after definitive chemoradiotherapy. We expect that t-PDT will be a useful therapy to improve the prognosis of patients with localized ESCC in the future.

## Supporting Information

File S1 **Supporting data. Data S1, The fluorescence intensity of talaporfin sodium in cultured ESCC cells.** (A) TE-5, (B) TE-8, (C) TE-10, (D) TE-11, (E) TE-5R, (F) TE-11R, Comp-APC-A: Compensated-Allophycocyanin (APC)-Area. **Data S2, Cytotoxic effect of t-PDT on ESCC cells.** The number of viable cells after t-PDT was expressed as a percentage of unirradiated control cells. (A) TE-5, (B) TE-8, (C) TE-10, (D) TE-11, (E) TE-5R, (F) TE-11R. **Data S3, Flow cytometric analysis with Annexin V/Propidium iodide double-staining.** Annexin V and Propidium iodide (PI) is detected by FITC and phycoerythrin (PE), respectively. **Data S4, Xenograft tumor size.** Each table represents the tumor volume (upper table) and the response rate (lower table).
(XLSX)
